# Housework as an Exercise

**Published:** 1892-06

**Authors:** 


### HOUSEWORK AS AN EXERCISE.

To keep the complexion and spirits good, to preserve grace, strength, and agility of motion, there is no gymnasium so valuable, no exercise more beneficent in result, than sweeping, dusting, making beds, washing dishes, and the polishing of brass and silver. One year of such muscular effort within doors, together with regular exercise in open air, will do more for a woman's complexion than all the lotions and pomades that were ever invented. Perhaps the reason why housework does so much more for women than games, is the fact that exercise which is immediately productive, cheers the spirit. It gives women courage to go on living and makes things seem really worth while.—*Medical Record.*

### EXERCISE.

Man and woman need exercise, and if a proper amount of exercise is not taken, not only do the muscles become weak and flabby, but the functions of every organ and the soundness of every tissue must suffer.

There is imperfect elimination of waste matters, the muscles and internal organs become encumbered with superfluous fat, the heart becomes weak, the lungs are never thoroughly emptied, and gradually lose their elasticity ; appetite dwindles to vanishing point, digestion become imperfect, often causing dyspepsia, and the joy and brightness of health give place to incapacity for either work or pleasure, irritability, and "leaden-eyed despair."

